# A Systematic Review on the Possible Relationship Between Bilingualism, Cognitive Decline, and the Onset of Dementia

**DOI:** 10.3390/bs9070081

**Published:** 2019-07-23

**Authors:** Maurits Van den Noort, Katrien Vermeire, Peggy Bosch, Heike Staudte, Trudy Krajenbrink, Lars Jaswetz, Esli Struys, Sujung Yeo, Pia Barisch, Benoît Perriard, Sook-Hyun Lee, Sabina Lim

**Affiliations:** 1 Research Group of Pain and Neuroscience, Kyung Hee University, Seoul 130-701, Korea; 2 Brussels Institute for Applied Linguistics, Vrije Universiteit Brussel, 1050 Brussels, Belgium; 3 Department of Communication Sciences and Disorders, Long Island University (LIU) Brooklyn, Brooklyn, NY 11201, USA; 4 Psychiatric Research Group, LVR-Klinik Bedburg-Hau, 47511 Bedburg-Hau, Germany; 5 Donders Institute for Brain, Cognition, and Behaviour, Radboud University, 6525 Nijmegen, The Netherlands; 6 Department of Medicine, Neurology, University of Fribourg, 1700 Fribourg, Switzerland; 7 Behavioural Science Institute, Radboud University, 6525 Nijmegen, The Netherlands; 8 College of Oriental Medicine, Sang Ji University, Wonju 26339, Korea; 9 Department of Psychology, Ruhr University Bochum, 44801 Bochum, Germany

**Keywords:** aging, bilingualism, cognitive decline, cognitive reserve hypothesis, dementia, onset

## Abstract

A systematic review was conducted to investigate whether bilingualism has a protective effect against cognitive decline in aging and can protect against dementia. We searched the Medline, ScienceDirect, Scopus, and ERIC databases with a cut-off date of 31 March 2019, thereby following the guidelines of the Preferred Reporting Items for Systematic Reviews and Meta-analysis (PRISMA) protocol. Our search resulted in 34 eligible studies. Mixed results were found with respect to the protective effect of bilingualism against cognitive decline. Several studies showed a protective effect whereas other studies failed to find it. Moreover, evidence for a delay of the onset of dementia of between 4 and 5.5 years in bilingual individuals compared to monolinguals was found in several studies, but not in all. Methodological differences in the set-up of the studies seem to explain these mixed results. Lifelong bilingualism is a complex individual process, and many factors seem to influence this and need to be further investigated. This can be best achieved through large longitudinal studies with objective behavioral and neuroimaging measurements. In conclusion, although some evidence was found for a cognitive reserve-enhancing effect of lifelong bilingualism and protection against dementia, to date, no firm conclusions can be drawn.

## 1. Introduction

The world population is aging, and this fact will have a large impact on healthcare systems [1]. As a result, during the last decade, we have seen a rise in the number of individuals suffering from major neurocognitive disorders, such as dementia [2]. Due to this increase in the absolute number of patients with dementia, the social and healthcare costs in society are high; the global societal costs of dementia are estimated to be around 818 billion US Dollars or 1.09% of the worldwide Gross Domestic Product [3], and these costs are expected to expand in the years to come [3]. Thus, the exact factors underlying this and the factors that may delay or prevent the onset of dementia are increasingly the subjects of investigation [4].

Differences between individuals in the way they are affected by brain damage or pathology have been reported in the literature. Individuals with more cognitive reserve were found to function better after the same amount of brain damage or pathology compared to individuals with less cognitive reserve [5]; this phenomenon is referred to as the “cognitive reserve hypothesis” [6,7]. This hypothesis refers to differences in coping with brain impairment as a result of differences in cognitive processes due to differences in lifetime experiences and intellectual activities and contexts [8]. Several factors were found to contribute positively to cognitive reserve: having a higher level of education [9], performing complex occupations [10], and having cognitively stimulating leisure activities [11]. Previous research, indeed, found a relationship between these cognitive reserve-enhancing factors and a reduced risk of dementia [12]. Interestingly, a suggestion has been put forward that bilingualism may be one of those cognitive reserve-enhancing factors [13].

Nowadays, bilingualism is widespread, and the majority of the world population has been estimated to be bilingual [14]; moreover, this number is expected to increase further in the years to come [15] due to increased migration patterns, the development of the internet, and international travel for work or tourism [16]. Bilingualism was found to have an influence on cognition beyond the linguistic domain [16], particularly executive functioning [17,18]. For instance, the fact that bilingual speakers constantly use both languages was found to improve aspects of attention and cognitive control [19,20]. Therefore, bilingualism might be contributing to cognitive reserve and, as a result, lead to protection against or a delay in the onset of major neurocognitive disorders, such as dementia.

In addition to behavioral studies, neuroscience research has also focused on the possible link between bilingualism and cognitive decline at a neural level. Previous neuroscience studies have revealed that particularly the prefrontal and posterior (mainly parietal) areas are involved in executive functioning [21], and that the evidence of specificity and commonality of executive processes at the cognitive level, as proposed by Miyake and colleagues [22], has been confirmed at the cerebral level. With respect to the main brain areas affected by dementia, it is known that an early stage of the disease, neurons and their connections in parts of the brain involved in memory, including the entorhinal cortex [23] and hippocampus [24], are destroyed [25]. At later stages of the disease, areas in the cerebral cortex [26] (e.g., known to be involved in language, reasoning, and social behavior [27]) are affected. It is thus possible that some of these brain areas may be involved in the cognitive reserve-enhancing effect of lifelong bilingualism.

The aim of the present study is to provide an overview of the studies that have been conducted in the field of bilingualism and the protection of individuals against cognitive decline. Moreover, we are particularly interested in whether or not bilingualism can delay the onset of dementia. In a society with a growing number of old adults, finding factors that may protect individuals or delay cognitive decline and major neurocognitive disorders, such as dementia, is increasingly important [4]. We expect to find that as a result of the daily use of two languages, resulting in improved attention and cognitive control skills [19,20], bilingualism can protect individuals against cognitive decline in old age. Secondly, we hypothesize that as a result of more (neural) cognitive reserve [13], bilingualism can delay the onset of dementia.

## 2. Materials and Methods

### 2.1. Search Strategies

We conducted a systematic review on bilingualism and the cognitive reserve hypothesis [6,7]. We were interested in whether or not bilingualism can protect individuals against cognitive decline, and we were especially interested in whether or not bilingualism can delay the onset of dementia. In this study, we searched the Medline (https://www.ncbi.nlm.nih.gov/pubmed/), ScienceDirect (https://www.sciencedirect.com/), Scopus (https://www.elsevier.com/solutions/scopus), and ERIC (https://eric.ed.gov/) databases with a cut-off date of March 31, 2019. We followed the guidelines of the Preferred Reporting Items for Systematic Reviews and Meta-analysis (PRISMA) protocol in our review study [28]. We used the following combinations of keywords in our search: “bilingualism” AND “cognitive reserve”, “bilingualism” AND “cognitive decline”, “bilingualism” AND “Alzheimer’s disease”, “bilingualism” AND “dementia”, “bilinguals” AND “cognitive decline” and “bilinguals” AND “Alzheimer’s disease”. Only full data papers or review papers were selected for further analysis; commentary papers and case studies were excluded.

### 2.2. Study Selection and Data Extraction

Four authors (M.N., K.V., P.B., and H.S.) independently searched the Medline, ScienceDirect, Scopus, and ERIC databases whereas four different authors (T.K., L.J., E.S., and S.Y.) independently performed the study selection and data extraction. The selection of relevant studies was conducted based on previously determined inclusion and exclusion criteria. To be considered for inclusion, the study had to be published in a peer-review format. The extracted data consisted of the following information: the journal in which the study had been published, the authors and the title of the study, the publication year, the number of participants that had been entered into the study, the languages that were involved, the age of second language acquisition, the level of education (if available), and information about the exact methodology that had been used in the study. Note that in the present systematic review, we used a more inclusive definition of (neural) cognitive reserve, meaning that also patient studies without direct measures of brain structure (that would determine the degree of damage or pathology) were included (we refer to the Discussion for a more detailed discussion of this issue). In cases of disagreement, four different authors (P.B.A., B.P., S.H.L., and S.L.) were asked to evaluate the study in question for inclusion in this review. In all cases, consensus could be reached among all twelve authors.

## 3. Results

**Figure 1:**
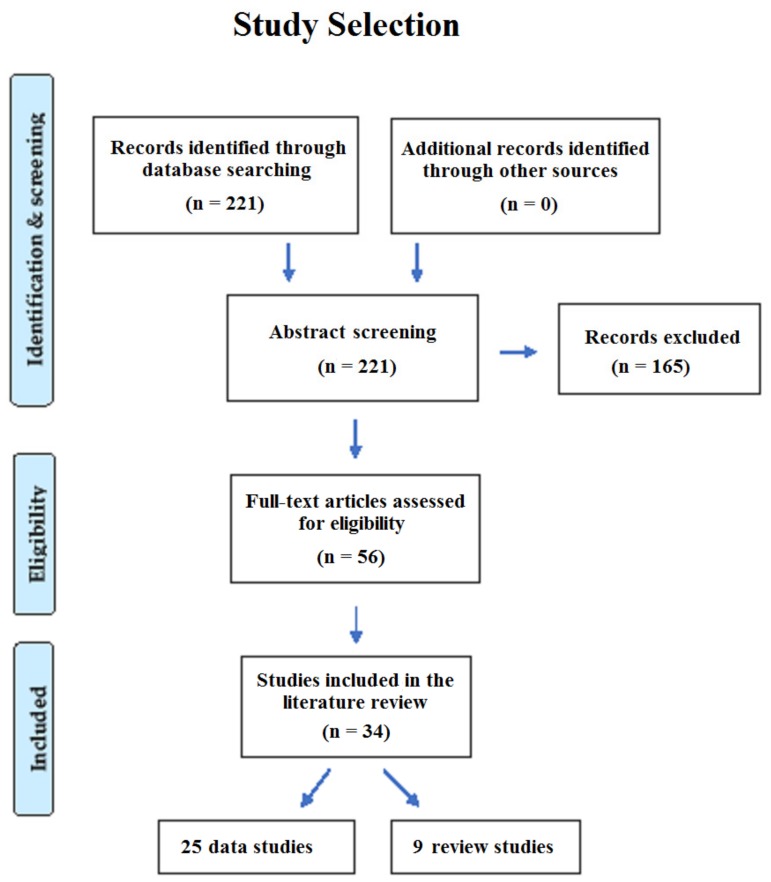
Overview of the selection process for the studies included in this review.

As can be seen in Figure 1, our search resulted in 221 articles of which 56 articles were relevant. Thirty-four of those satisfied the inclusion criteria of our study and were, thus, eligible for inclusion in this review. Of the 34 studies, 25 were original studies [13,29,30,31,32,33,34,35,36,37,38,39,40,41,42,43,44,45,46,47,48,49,50,51,52] and 9 were review studies [53,54,55,56,57,58,59,60,61]. Ten studies investigated the relationship between bilingualism and cognitive decline in healthy individuals. As can be seen in Table 1b, we found eight original studies [29,32,34,36,44,48,50,51] (Table 1a) and two review studies [53,54]. In total, 4946 bilingual subjects and 4524 monolingual subjects participated in the studies on the relationship between bilingualism and cognitive decline. Twenty-four of the 34 studies investigated the relationship between bilingualism and the onset of dementia: 17 original studies (Table 2a) [13,30,31,33,35,36,37,39,40,41,42,43,44,45,47,49,51] and 7 review studies [55,56,57,58,59,60,61] (Table 2b). In total, 2794 bilingual subjects and 4207 monolingual subjects participated in the studies on the relationship between bilingualism and the onset of dementia.

*Please note that in order not to count one study twice, we decided to list the review study by Bialystok and colleagues [53] here.

**Figure 2:**
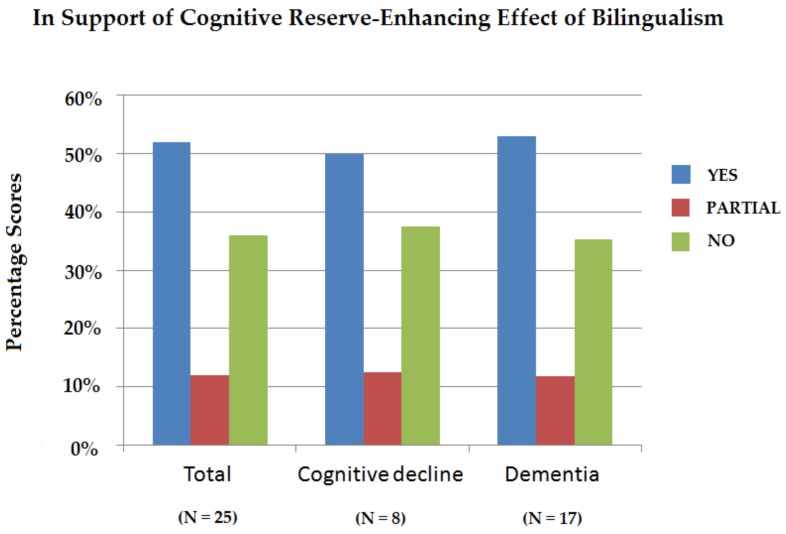
Overview of number of original studies (in percentages) in support of, partially in support of, or against a cognitive reserve-enhancing effect of bilingualism specified for the total number of original studies, for the number of original studies focusing on cognitive decline in healthy individuals, and for the number of original studies focusing on dementia.

As can be seen in Figure 2, with respect to the total number of original studies, in 52.00% (n = 13) of these studies evidence was found in favor of a cognitive reserve-enhancing effect of bilingualism, in 12.00% (n = 3) partial evidence was found, and in 36.00% (n = 9) evidence against a cognitive reserve-enhancing effect of bilingualism was found. If we take a closer look at the studies focusing on cognitive decline in healthy individuals, the results are slightly different. In half of the original studies (50.00%) (n = 4), evidence was found in favor of a cognitive reserve-enhancing effect of bilingualism, in 12.50% (n = 1), partial evidence was found, and in 37.50% (n = 3), evidence against a cognitive reserve-enhancing effect of bilingualism was found. Finally, the results of the studies focusing on dementia show the most positive results in favor of the existence of a cognitive reserve-enhancing effect of bilingualism. In 52.94% (n = 9) of the original studies, evidence was found in favor of a cognitive reserve-enhancing effect of bilingualism, in 11.76% (n = 2), partial evidence was found, and in 35.30% (n = 6), evidence against a cognitive reserve-enhancing effect of bilingualism was found.

### 3.1. Protection against Cognitive Decline

We first present the results that were found in studies investigating the relationship between bilingualism and cognitive decline in healthy individuals (Table 1a). Kavé and colleagues [29] conducted a follow-up study on older, healthy individuals who were first tested between 1989 and 1992. In their study, a cognitive screening [62,63] of bilinguals, trilinguals, and individuals who spoke more than three languages took place, and the test results were compared with the previous test results. The number of languages spoken partly predicted the cognitive test scores at old age. This was still the case when other variables, such as age, gender, place of birth, age at immigration, or education, were taken into account. Moreover, the study revealed that multilingualism was a significant predictor of cognitive functioning. Interestingly, the individuals who were better in their foreign language than in their native language, on average, showed better results than the individuals whose native language was their best language. Bak and colleagues [38] conducted a follow-up study on older adults. All participants were re-tested on a large battery of psychological tests [64,65,66] in order to test general fluid-type intelligence, memory, speed of information processing, verbal reasoning, vocabulary, reading, and verbal fluency capacity of the individuals, and these results were compared with the results of the first testing when the participants were 11 years old. The researchers were especially interested in whether or not the previously reported cognitive reserve-enhancing effect of bilingualism might be explained by a difference in childhood intelligence from the beginning. They found that this was not the case. Moreover, they found that bilingualism contributed to cognitive reserve, regardless of age of second language acquisition. The beneficial effect of bilingualism was visible in both individuals who acquired the second language as a child and individuals who acquired the second language in adulthood (However, here, in contrast to Bak and colleagues [38], it is important to add that not all researchers consider their study results as support for the cognitive reserve-enhancing effect of bilingualism. Paap and colleagues [67] (see page 311), for instance, consider their results rather as no more than “partial” evidence because these beneficial effects were not found on all experimental tasks, the effects were not very large nor very consistent, and were apparently achieved and maintained without the need to remain actively bilingual). Ihle and colleagues [46] conducted a study on older adults in Switzerland. They used psychometric tests of verbal abilities, basic processing speed, and cognitive flexibility [68,69] and interviewed all participants. They found that speaking different languages on a regular basis may contribute to cognitive reserve in old age, yet this may be influenced by individual differences.

In addition to behavioral assessments, other measurement techniques are increasingly being used. Estanga and colleagues [48], for instance, conducted a neurobiological study on healthy, middle-aged individuals, analyzing Alzheimer’s disease (AD) biomarkers in cerebrospinal fluid (e.g., amyloid beta (Aβ) 1‒42, total-tau, and phosphorylated-tau, as well as ratios of total-tau/Aβ_1‒42_ and phosporylated-tau/Aβ_1‒42_). The researchers used a wide range of neuropsychological tests [63,65,69,70,71,72,73,74,75,76] to assess their monolinguals, early bilinguals (who acquired their second language before the age of six), and late bilinguals (who acquired their second language after the age of six). A moderation effect was found for bilingualism on the relationship between age and cerebrospinal fluid AD biomarkers and on the relationship between age and executive functioning, supporting the cognitive reserve hypothesis. Moreover, Anderson and colleagues [50] conducted a diffusion tensor imaging study on bilingual and monolingual healthy older adults, investigating white matter integrity in the brain. The results showed that after controlling and matching for confounds (e.g., intelligence, mini-mental state scores, and demographic variables), a greater axial diffusivity in the left superior longitudinal fasciculus was found in bilinguals compared to monolinguals. The finding of greater white matter integrity in bilinguals compared to monolinguals supports the hypothesis of a cognitive reserve-enhancing effect of bilingualism at a neural level. As can be seen in Table 1b, this is also the conclusion that was drawn in two recent review studies [53,54]. Bialystok and colleagues [53] conclude in their review study on the protective effects of bilingualism in aging that bilingualism is a potent source of cognitive reserve. Moreover, Quinteros Baumgart and Billick [54] found evidence for a cognitive reserve-enhancing effect of lifelong bilingualism and multilingualism; however, the authors point to the issue that several factors, like immigration and personal experiences, seem to affect the extent of this effect.

In contrast to the previously discussed studies, not all studies found evidence for a protective effect of bilingualism against cognitive decline. Crane and colleagues [32], for instance, investigated bilingual (Japanese-American) older adults, and they measured cognitive functioning [77]. Their sample consisted of three subgroups: individuals that neither spoke nor read Japanese, individuals that only spoke Japanese, and individuals that both spoke and read Japanese. The authors found that the use of neither spoken nor written Japanese in midlife led to a reduction in cognitive decline in later life, showing no evidence for a cognitive reserve-enhancing effect of lifelong bilingualism. Similar results were found by Kousaie and Phillips [34] who also reported no evidence for a cognitive reserve-enhancing effect of lifelong bilingualism. No differences in interference scores [70] were found between the group of healthy older bilingual adults and the group of healthy older monolingual adults. This was also what Mukadam and colleagues [51] found in their Australian longitudinal study with cognitive functioning tests [63,66,71,72] on older individuals. Moreover, they discovered that education rather than bilingualism was a predictor of the cognitive functioning score. Based on their results, Mukadam and colleagues [51] state that bilingualism is a complex phenomenon and when bilingualism is not the result of greater educational attainment, it does not always protect older individuals from cognitive decline. Finally, in line with this statement and based on their own study findings, Kousaie and Phillips [34] question the robustness and/or specificity of the cognitive reserve-enhancing effect of lifelong bilingualism.

### 3.2. Delaying the Onset of Dementia

So far, we have presented studies that investigated the relationship between bilingualism and cognitive decline in healthy individuals. In the next part of our paper, we will focus on individuals that suffer from dementia. The questions that we are interested in are: Is bilingualism a cognitive reserve factor? Can bilingualism delay the onset of dementia in bilingual older adults? As can be seen in Table 2a, Bialystok and colleagues [13] investigated the potential cognitive reserve-enhancing effect of lifelong bilingualism on maintaining cognitive functioning and delaying the onset of symptoms of dementia in older adults. They investigated bilingual and monolingual patients with dementia. The symptoms of dementia appeared four years later in the sample of bilingual older adults as compared to the sample of monolingual older adults. Moreover, the results of cognitive screening [63] over the four years prior to the diagnosis of dementia showed similar cognitive decline scores for both groups. Taken together, evidence was found for the cognitive reserve hypothesis and for the cognitive reserve-enhancing effect of lifelong bilingualism. In line with the previous study, Craik and colleagues [31] investigated a group of patients with probable AD. They found that the bilingual patient group had been diagnosed, on average, 4.3 years later than the monolingual patient group. Moreover, the bilingual patients had reported the onset of symptoms, on average, 5.1 years later than the monolingual patient group. The results found by Craik and colleagues [31] confirmed the previous findings by Bialystok and colleagues [13], supporting the idea of a cognitive reserve-enhancing effect of lifelong bilingualism. This is also what Woumans and colleagues [44] found in their study on patients with AD. The results revealed that the bilingual patients showed a significant delay of 4.6 years in clinical manifestation of AD and 4.8 years in diagnosis compared to the monolingual patients. In addition, similar results were obtained by Alladi and colleagues [37] in a study on middle-aged to older-aged patients with dementia. They found that the bilingual participants developed dementia 4.5 years later than the monolingual participants. Importantly, this finding cannot be explained by other confounding factors, such as level of education, gender, professional background, and place of living (urban versus rural) (Although Paap and colleagues [67] (see page 312) criticize the use of samples of individuals who present themselves at clinics, as was used in the Alladi et al. [37] study, because the language groups in that study differed dramatically in other ways: the bilinguals were better educated, were from higher skill occupations, and included a higher proportion of men and a higher proportion from urban populations [67]. On the other hand, exactly these confounding factors were controlled for and could not explain the differences that were found). The important contribution of the study by Alladi and colleagues [37] is that they investigated five types of dementia, AD [79], dementia with Lewy bodies [80], frontotemporal dementias [80], vascular dementia [80], and mixed dementia [79], instead of dementia in general, which is especially important because these types have their own trajectories of cognitive decline [80]. Significant delays in onset age of dementia were found for several types of dementia: AD, dementia with Lewy bodies, and frontotemporal dementias. However, the delays did not reach significance in all types of dementia; no significant delays in the onset age of dementia were found for vascular dementia and mixed dementia. Furthermore, Gollan and colleagues [33] tested bilingual patients with probable AD by using both objective [71] and subjective measures of second language proficiency. Their results support the hypothesis that lifelong bilingualism delays the onset of AD. An association was found between higher degrees of bilingualism and increasingly later age-of-diagnosis of AD, but this was only found to be the case for the patients with a low education level. Moreover, only the results obtained with objective second language proficiency measurements were found to be a reliable predictor. In a study by Bialystok and colleagues [39], the participants were assessed using several cognitive functioning instruments [63,81,82]. In the AD group, a delay of 7.3 years in the onset of AD in comparison with the monolinguals was found; moreover, these results could not be explained by differences in lifestyle variables between the bilinguals and the monolinguals. In a recent study, Zheng and colleagues [52] investigated older adults with probable AD. The sample consisted of Cantonese/Mandarin bilinguals, Cantonese monolinguals, and Mandarin monolinguals. They used a structured interview and a cognitive screening instrument [63] for the assessments. The results of the study showed that the Cantonese/Mandarin bilinguals had a delay in the onset of AD of 5.5 years compared to the monolinguals; moreover, the bilinguals were found to be older at their first clinic visit compared to the monolinguals. Taken together, the patient studies on dementia that were done using behavioral measurements clearly showed evidence in favor of a cognitive reserve-enhancing effect of lifelong bilingualism on maintaining cognitive functioning and delaying the onset of symptoms of dementia by, on average, 4 to 5.5 years in older bilingual patients as compared to the monolingual patients [13,31,39,44,52]. As can be seen in Table 2b, this is also the conclusion that was drawn in several recent review studies [53,55,56,58].

The cognitive reserve-enhancing effect of lifelong bilingualism was also confirmed in neuroscience research [36,47,49]. Schweizer and colleagues [36] analyzed computed tomography (CT) data of bilingual and monolingual older adults with probable AD. They found substantially greater amounts of brain atrophy in areas that are traditionally used to diagnose AD clinically in bilingual patients than in monolingual patients. Their results indicate that greater amounts of neuropathology are needed in bilingual patients with probable AD than in monolingual patients with probable AD before the clinical symptoms of the disease become visible. Furthermore, Kowoll and colleagues [47] investigated bilingual and monolingual older adults who had been diagnosed with either mild cognitive impairment or with early stage AD in a fludeoxyglucose (^18^F) positron emission tomography (PET) study. The results showed that bilingualism is likely to contribute to cognitive reserve. Bilingual patients showed substantially greater impairment of glucose uptake in frontotemporal regions, in parietal regions, and in the left cerebellum than monolingual patients, indicating that in the early stages of AD, bilingual patients can compensate for more severe cerebral impairments than monolingual patients [47]. Perani and colleagues [49] conducted a fludeoxyglucose (^18^F) PET study as well in their investigation of brain metabolism and neural connectivity in bilingual and monolingual patients with probable AD. The results showed an increased connectivity in the executive control and the default mode networks in the bilingual patients as compared to the monolingual patients. Moreover, the study revealed that the degree of lifelong bilingualism (i.e., high, moderate, or low use) was significantly correlated to functional modulations in crucial neural networks. Perani and colleagues [49] interpret their neuroimaging results as evidence for both neural reserve and compensatory mechanisms in bilingual patients with probable AD, confirming the results found in previous studies on the cognitive reserve-enhancing effect of lifelong bilingualism [13,31,44] and the conclusions that were drawn in several recent review studies on the contribution of bilingualism to cognitive reserve on a neural level (Table 2b) [56,57,58,60].

However, not all studies found evidence for a cognitive reserve-enhancing effect of lifelong bilingualism in older adults. Clare and colleagues [45], for instance, investigated patients with probable AD on a whole test battery of executive functioning tasks. Their results showed no advantage in cognitive control tasks for the bilinguals. Only the fact that the bilingual patients came later to the attention services than the monolingual patients might be indirect support for some delay in AD, but if so, the results are less convincing than in previous studies. Moreover, Chertkow and colleagues [30] investigated patients with probable AD. Their results showed a protective effect of bilingualism in native Canadians whose first language was French, but not in those whose first language was English. In addition, a protective effect of bilingualism was found in immigrants to Canada. Overall and in individual groups, speaking more than three languages was found to have a protective effect, but this was not (always) the case for speaking two languages. Yeung and colleagues [40] used a structured interview and a cognitive screening instrument [83] in their assessments. They found no association between being bilingual and having dementia in the analysis of a large group of older adults. Moreover, for the individuals who were cognitively healthy at the first time of measurement, no association was found between speaking more than one language and dementia at the second time of measurement five years later. Zahodne and colleagues [41] studied bilingual and monolingual Spanish-speaking immigrants on various cognitive function tasks [84,85,86,87,88]. Although bilingual older adults were found to have better memory and executive function skills than monolinguals at baseline, no protective effect of bilingualism was found. In other words, bilingualism did not alter cognitive decline or protect against dementia. Kowoll and colleagues [42] found no evidence for a cognitive reserve-enhancing effect of lifelong bilingualism in their study with a large test battery of cognitive functioning tests [42,63,69,84,89,90,91] on patients with mild cognitive impairment, patients with AD, and healthy controls. Interestingly, the dominant language was discovered to be affected first in bilingual patients with mild cognitive impairment. Moreover, deficits of the second language appear later in bilingual patients suffering from AD. Lawton and colleagues [43] used various cognitive functioning tests [83,92,93,94] as well and found no support for the hypothesis that lifelong bilingualism delays the onset of AD in their study on older Hispanic Americans with AD. Finally, Sanders and colleagues [35] conducted a study on a large group of older bilingual and monolingual adults. They found no evidence for a relationship between lifelong bilingualism and the onset of AD. Surprisingly, when education was further assessed, evidence in the opposite direction was found: highly educated bilinguals might be at increased risk for dementia and or AD. In conclusion, to date, the results of the research on the existence of a possible cognitive (neural) reserve-enhancing effect of lifelong bilingualism in older adults are not straightforward. Methodological differences (and weaknesses) in the set-up of the studies make comparisons and interpretations of the results across different research groups difficult, which was also the conclusion that was drawn in two recent review studies (Table 2b) [59,61].

## 4. Discussion

A systematic review was conducted to provide an overview of studies that had been conducted in the field of bilingualism and the protection of individuals against cognitive decline. We were particularly interested in whether or not bilingualism can delay the onset of dementia. In a society with a growing number of old adults, finding factors that may protect individuals against or delay cognitive decline and dementia is increasingly important [4].

Firstly, we expected to find that bilingualism can protect individuals against cognitive decline. The results showed that, indeed, evidence exists for a cognitive reserve-enhancing effect of lifelong bilingualism [29,38,48,50]; this evidence was found to exist in both individuals who acquired the second language as a child and in individuals who acquired the second language as an adult [38]. This cognitive reserve-enhancing effect was even found to be larger for trilingualism and was found to be the highest for individuals who spoke four or more foreign languages [29]. One could argue that this finding could perhaps be explained by a difference in childhood intelligence between the monolinguals and the bilinguals; however, even after controlling for childhood intelligence, the cognitive reserve-enhancing effect of lifelong bilingualism remained [38]. In addition, further evidence comes from neuroscience research. Estanga and colleagues [48], for instance, found in their neurobiological study on healthy, middle-aged individuals, an association between (early) bilingualism and the presence of AD biomarkers in cerebrospinal fluid. Early bilinguals showed lower cerebrospinal fluid t-tau levels (which is an AD biomarker) than monolinguals and had a lower prevalence of preclinical AD (according to the criteria of the National Institute on Aging-Alzheimer’s Association classification [95]), proving the cognitive (neural) reserve-enhancing effect of bilingualism. Moreover, Anderson and colleagues [50] conducted a diffusion tensor imaging study and found a greater axial diffusivity in the left superior longitudinal fasciculus in bilingual older adults compared to monolingual older adults. This finding remained after controlling for important mediating background variables, such as gender, age, education, verbal and spatial intelligence, visual attention and task switching, and cognitive screening. Anderson and colleagues [50] conclude that the greater white matter integrity in the axial diffusivity in bilinguals might contribute to (neural) cognitive reserve in bilinguals, facilitating communication between brain areas that are otherwise suffering from deterioration [50]. The idea is that the combination of white matter integrity [96] and functional reorganization in the brain as a result of lifelong bilingualism [97] both contribute to extra (neural) cognitive reserve in bilinguals compared to monolinguals. However, not all studies found evidence for a protective effect of bilingualism against cognitive decline [32,51]. Crane and colleagues [32], for instance, found that neither the use of spoken nor written Japanese in midlife led to a reduction in cognitive decline in later life. Mukadam and colleagues [51] conclude that when bilingualism is not the result of greater educational attainment, it does not always protect older individuals from cognitive decline. Taken together, the results on the cognitive reserve-enhancing effect of lifelong bilingualism in aging are not straightforward. For half of the original studies, evidence was found in favor of a cognitive reserve-enhancing effect of bilingualism, in 12.50%, partial evidence was found, and in 37.50%, evidence against a cognitive reserve-enhancing effect of bilingualism was found. The contribution of bilingualism to cognitive reserve in aging seems to be stronger for lifelong multilingualism than for lifelong bilingualism [29]; however, many factors seem to affect this [51]; as a result, the picture is a complex picture, and perhaps the cognitive reserve-enhancing effect of lifelong bilingualism in aging [53,54] is not a robust and universal phenomenon at all [34,46].

Secondly, we hypothesized that bilingualism can delay the onset of dementia. Patient studies on dementia showed evidence in favor of delaying the onset of symptoms of dementia, on average, for 4 to 5.5 years in older bilingual patients as compared to monolingual patients [13,31,37,39,44,52]. The behavioral studies in which large samples of patients with dementia are studied, in contrast to bilingualism research on cognitive control in healthy young- to middle-aged subjects [16], showed a cognitive reserve-enhancing effect of lifelong bilingualism on maintaining cognitive functioning. Further support for (neural) cognitive reserve as a result of lifelong bilingualism was found in neuroscience research [47,49]; an increased connectivity in the executive control and the default mode networks was found in the bilingual patients as compared to the monolingual patients [49], proving that bilingualism is likely to contribute to cognitive reserve [47]. Additional evidence comes from a study by Schweizer and colleagues [36] who analyzed a number of linear measurements of brain atrophy in their CT study. They found supporting data that greater amounts of neuropathology are needed before the clinical symptoms of AD become visible in bilinguals. However, in contrast to the majority of studies [53,58], not all studies found a cognitive reserve-enhancing effect of lifelong bilingualism. In some studies, only partial evidence was found [30,33]. According to Chertkow and colleagues [30], a cognitive reserve-enhancing effect exists for lifelong multilingualism, but not for lifelong bilingualism per se. Moreover, Gollan and colleagues [33] did find the cognitive reserve-enhancing effect of lifelong bilingualism, but only in patients with AD with a low education level. Other studies failed to find any evidence in favor of the cognitive reserve-enhancing effects of bilingualism at all. Clare and colleagues [45], for instance, found no advantages in executive control in bilinguals. Sanders and colleagues [35] found no statistically significant association between non-native speakers of English and dementia or between non-native speakers of English and AD. Similar results were reported by Yeung and colleagues [40]; no association was found between speaking more than one language and dementia. Moreover, Zahodne and colleagues [41] failed to find a cognitive reserve-enhancing effect of lifelong bilingualism. Bilingualism was found not to alter cognitive decline or protect against dementia. Finally, the results collected by Lawton and colleagues [43] and by Kowoll and colleagues [42] did not support its existence either. In sum, although in 53% of the original studies, evidence was found in favor of a cognitive reserve-enhancing effect of bilingualism, in 12% of the original studies, only partial evidence was found, and in 35% of the original studies, evidence against a cognitive reserve-enhancing effect of bilingualism was found. Regarding these general results, Paap and colleagues [67] stress that sometimes significant differences emerge only when other confounding variables are taken into account; moreover, they argue that some of the reported results, like the results reported by Woumans et al. [44], seem convincing at first sight, but a deeper look at the results reveal a less convincing picture [67] (see page 312). Paap and colleagues furthermore point towards the methodological issue of using non-sensitive experimental tests. Given that the frequently used MMSE [63] in research on the relationship between bilingualism and dementia is known for its lack of sensitivity to mild cognitive impairment [98], it is not surprising that the subgroups (even the high occupation monolinguals) do not initially differ in their MMSE scores due to a ceiling effect [98,99].

**Figure 3:**
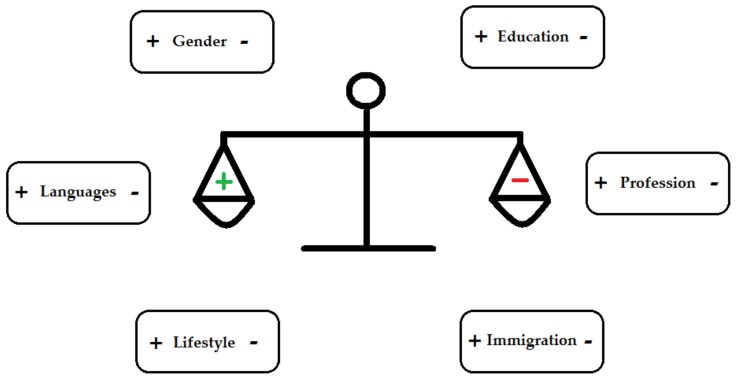
Factors that seem to affect the cognitive reserve-enhancing effect of lifelong bilingualism.

Why are the results from studies on the relationship between bilingualism and cognitive reserve and the onset of dementia so heterogeneous? As can be seen in Figure 3, six factors seem to affect the cognitive reserve-enhancing effect of lifelong bilingualism. First, monolinguals and bilinguals might differ in the level of education, with higher baseline scores in cognitive functioning and a better education in bilinguals [41,43,51]. This effect on cognitive reserve, though, can be in all directions (positive, neutral, or negative). In addition to a positive effect of education on cognitive reserve [100], an upper limit seems to exist on the extent to which reserve can function to delay dementia [33]; the effect can even go in the opposite direction: highly educated bilinguals might be at increased risk for dementia and/or AD [35]. A second factor that seems to affect the cognitive reserve-enhancing effect of lifelong bilingualism is immigration [54]. Immigrant families generally are disproportionally poorer [101], and previous research has shown that children in poorer households receive less language input, the language input is less varied, and the language input is less positive [54]. A third factor that seems to affect the cognitive reserve-enhancing effect of lifelong bilingualism is the kind of language one speaks [30]. Chertkow and colleagues, for instance, found a protective effect of bilingualism in native Canadians whose first language was French, but not in those whose first language was English [30]. A fourth factor is lifestyle (e.g., social activity, physical activity, smoking, alcohol consumption, or diet) [102]. Reports in the literature suggest that aspects of life experience, for instance, engagement in leisure activities, results in functionally more efficient cognitive networks [102,103]. A fifth factor mediating cognitive reserve factor is profession [104]. Previous research showed that low-complexity occupations were found to be risk factors for cognitive decline in old age [105] while complex intellectual professions were found to have positive effects on cognitive functioning of older workers [10]. Last, but not least, gender seems to be a mediating cognitive reserve factor [106]. Poorer cognitive profiles were found in female patients than in male patients at the same stage of AD [107]. On the other hand, we must stress that previous research found evidence for the cognitive reserve-enhancing effect of lifelong bilingualism [37] and a delay in the onset of dementia in bilinguals [37] after taking into account these possible confounding factors, like level of education, gender, professional background, place of living, or differences in lifestyle variables (e.g., smoking, alcohol consumption, physical activity, diet, or social activity) [37,39,53,108]. Moreover, in a comparative study, Ramakrishnan and colleagues [109] showed that the cognitive reserve-enhancing effects of bilingualism were stronger than the cognitive reserve-enhancing effects of education. In sum, results for these confounding effects are mixed (Figure 3): That is, which factors exist and are their influence positive or negative in relation to cognitive reserve? Thus, further research is needed.

### 4.1. Neuroscience Research

**Figure 4:**
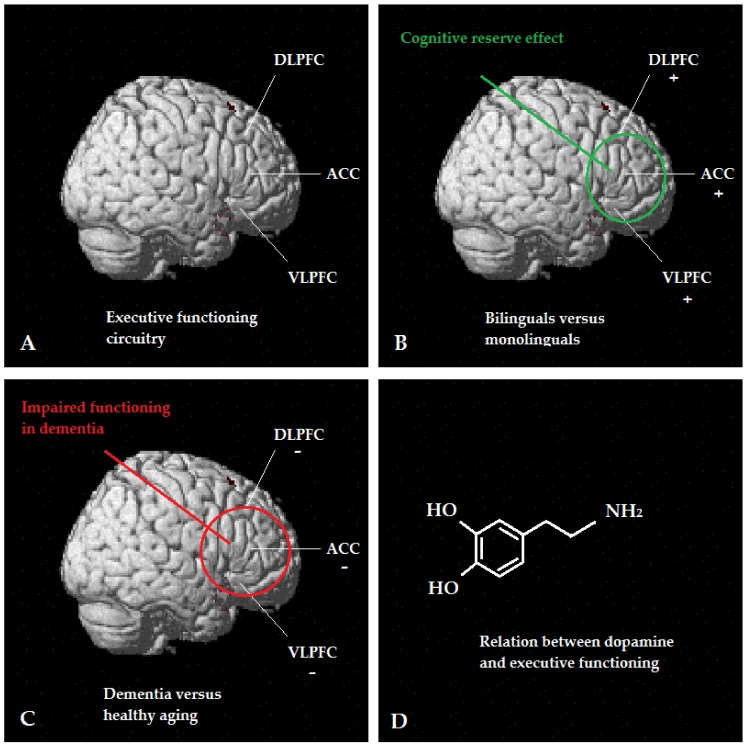
The protective effect of bilingualism against dementia works via the executive functioning circuitry (**A**). Bilinguals have a better-developed executive functioning circuitry (**B**) that becomes especially visible in neurocognitive disorders, such as dementia (**C**), in which exactly these areas, in addition to the memory circuitry, are affected by the disease. The functional and structural changes caused by lifelong bilingualism in the brain areas involved in executive functioning delays the onset of dementia, but it cannot stop the deterioration of the memory circuitry. In this protective and delaying effect of bilingualism, the neurotransmitter dopamine may play a key role in successfully regulating executive functioning (**D**). *Notes*. ACC = anterior cingulate cortex, DLPFC = dorsolateral prefrontal cortex, VLPFC = ventrolateral prefrontal cortex.

Neuroscience offers special tools and assessments to investigate the possible relationship between bilingualism and cognitive reserve. In contrast to behavioral studies, neuroscience makes possible direct investigation on aging individuals of neural, cellular, and molecular mechanisms in the brain that may underlie differences in behavioral results. A number of brain areas known to be involved in executive functioning circuits [110] seem to be involved in the cognitive reserve-enhancing effect of lifelong bilingualism: dorsolateral prefrontal cortex, ventrolateral prefrontal cortex, insula, anterior cingulate cortex, basal ganglia, thalamus, and posterior parietal cortex [111]. Moreover, previous research revealed that as a result of the active use of two languages (e.g., language switches, inhibition), bilinguals often outperform monolinguals in executive functioning skills [112]. Interestingly, the strength of frontal cortex activation was also found to be different for bilingual compared to monolingual healthy older adults during the performance of executive functioning tasks [113]. In line with these findings, Gold [56] suggested that the protective and delaying effect of bilingualism against the symptoms AD may work via the frontostriatal and frontoparietal executive functioning networks. Note that exactly these networks [114,115,116], in addition to the memory circuitry [117], are affected by dementia. The protective and delaying effect of bilingualism may operate via specific cellular and molecular mechanisms, affecting the neuronal metabolic functions, dynamic neuronal-glial interactions, vascular factors, myelin structure and neurochemical signaling [56]. In this protective effect of bilingualism, the neurotransmitter dopamine may play a special role [56] because it was found to play a key role in regulating executive functioning [110]. In previous neuroimaging research, a correlation was found to exist between executive control tasks and both dopamine receptor availability [118] and dynamic dopamine release [119]. Moreover, an optimal dopamine level for maximum attentional capacity [120] and inhibitory control [121] seems to exist. Note that attention and inhibitory control are vital for successfully performing cognitive tasks. Therefore, more brain research on the neurotransmitter dopamine in the protective and delaying effect of bilingualism is warranted; does lifelong bilingualism optimize dopamine levels? (See Figure 4)

The frontostriatal and frontoparietal executive functioning networks, and their underlying cellular and molecular mechanisms, need to be investigated further in order to gain insights into the cognitive reserve capacity of the aging brain and the possible contributing factor of lifelong bilingualism. In this respect, future neuroscience research with repetitive transcranial magnetic stimulation (rTMS) and with transcranial direct current stimulation (tDCS) seems to be promising for shedding more light on the possible protective effect of bilingualism against cognitive decline in the aging brain [122] because these non-invasive techniques make possible direct investigation of the frontostriatal and frontoparietal executive functioning networks in bilingual versus monolingual older adults; however, at the same time, recognizing the risks of brain stimulation in older adults is important in order to safely conduct these future brain stimulation studies [123]. Also, the use of the newly developed magnetic resonance elastography (MRE) technique [124] seems promising for use in future bilingual research on cognitive reserve, particularly because it makes possible almost real-time investigations of neural activity during executive functioning tasks in older bilingual and monolingual adults.

### 4.2. Limitations

Several methodological limitations exist in the research on the protective effect of bilingualism against cognitive decline and major neurocognitive disorders. Researchers point out that many factors (see Figure 3) can influence the cognitive reserve-enhancing effect of lifelong bilingualism [59]. Although this statement is correct, research on human subjects in real life also has natural methodological limitations. Controlling for all factors in real life is simply not possible because some of the factors may not have been identified yet, older adults do not live in laboratory settings (e.g., individual differences in the acquisition of a foreign language [125], the heterogeneity of dementia/AD [126], differences in social environment, etc.), and ethical rules place restriction on what researchers can and cannot do [127]. As a result, researchers can only attempt to take all known factors into account and control for those, as well as possible, interacting factors and/or make them the purpose of the investigation. For instance, the use of prospective studies, instead of retrospective studies, seems more promising for investigating any causative links between bilingualism and cognitive control, decline, and the onset of dementia [59]. Note that there is a discrepancy between the results found in prospective studies and the results found in retrospective studies [60,61]. In most prospective studies, no association between bilingualism and the delay of the onset of dementia was found while in the majority of retrospective studies an association between bilingualism and the delay of the onset of dementia seemed to exist [60,61]. According to Paap [128], there is little evidence that bilingualism protects against cognitive decline when the prospective studies are weighted more heavily. Nevertheless, when several confounding factors are taken into account [108], researchers have still found evidence in favor of a protective effect of bilingualism against cognitive decline [29] and in favor of bilingualism as a delaying factor in the onset of dementia [37,39].

Moreover, researchers investigating the protective effect of bilingualism against cognitive decline and major neurocognitive disorders often use the analysis of covariance (ANCOVA) in the statistical analysis of their results. However, as Paap and colleagues [129] discussed, a critical assumption of the ANCOVA is that the covariate and groups are independent [130]. When this is not the case then, the regression adjustment may either obscure part of the grouping effect (e.g., language effect) or produce spurious effects. Therefore, it is not possible to interpret the ANCOVA results when systematic differences in the covariate across monolingual and bilingual (patient) groups exist [129].

Another limitation of the present study (and of the research field in general) has to do with the concept “cognitive reserve”. There is a lack of consensus in the field regarding the exact definition of “(neural) cognitive reserve”, and what (neural) evidence is needed to determine its existence and degree. So far, most of the studies examining the relationship between bilingualism and cognitive functioning do not include measures of brain structure that would determine the degree of damage or pathology. That is, studies that are included compare bilingual to monolingual (patient) groups on measures of cognitive function (e.g., measures of executive functioning) or age-of-onset of dementia, but in most studies, we do not know if there are concomitant differences in brain structure. Even if neuroscience measurements are used it is still unclear what (neural) evidence is required to confirm the cognitive reserve hypothesis.

The present study makes clear that future studies on several methodological issues are warranted before any firm conclusions on the protective effect of bilingualism against cognitive decline and dementia can be drawn. For instance, future research is needed on the issue of early versus late bilingualism and how this affects functional connectivity in the brain [131]. In order to find those effects that protect against cognitive decline and that delay the onset of dementia in bilinguals, does it matter that one has acquired those two languages from birth onwards or later in life? Bak and colleagues [38] found in their study that the cognitive reserve-enhancing effect was visible, regardless of the age of acquisition of the foreign language (childhood versus adulthood). Other researchers stress the importance of actively using two languages on a daily basis in order to benefit from the cognitive-reserve effects of bilingualism [45]. Future research should address whether or not those cognitive reserve-enhancing effects are stronger for individuals who acquired the two languages at birth and who used those languages throughout their lives.

Moreover, future studies are needed to address whether or not the language family [132] matters with respect to the cognitive reserve-enhancing effect of lifelong bilingualism. Whether different effects are found for bilinguals who are bilingual in two languages from different language families (e.g., a West Germanic language versus a Romance language) compared to individuals who are bilingual in two languages from the same language family remains a question looking for an answer. One could argue that this might require different attention and executive functioning skills and, as such, might lead to more or less cognitive protection against and a delay of the onset of dementia.

The majority of studies on the cognitive reserve-enhancing effect of lifelong bilingualism so far have focused on AD (or dementia in general) [30,31,39,44,49]. However, lifelong bilingualism may also delay the onset age of other brain diseases, such as Parkinson’s disease [133]. So far, almost no research on this topic exists. In a study by Hindle and colleagues on 46 bilingual (Welsh/English) and 57 monolingual (English) speakers with Parkinson’s disease, no evidence for the cognitive reserve-enhancing effect of lifelong bilingualism was found [133]. Moreover, bilingualism might play a protective role for psychiatric diseases such as schizophrenia or depression. Unfortunately, to date, almost no research has been conducted on this topic, and it is too early to draw any firm conclusions [134]. However, the preliminary results collected so far indicate that in patients with schizophrenia, bilingualism might decrease social isolation and stigma and enhance job perspectives, but more research is needed [134].

Additionally, gender differences may exist in the cognitive protective effect of lifelong bilingualism, as previous research discovered gender differences in healthy elderly individuals and in patients with AD [135]. In a neuroimaging study on 282 patients with AD, a posterior temporo-parietal association in men and a frontal and limbic association in women were discovered. Men and women were found to differ with respect to the involvement of different brain networks [135]. Moreover, previous research revealed that gender differences exist in foreign language learning as female learners were found to outperform male learners in foreign language writing and speaking [136]. In addition, gender differences exist in the prevalence of dementia (including AD) [137]. Surprisingly, almost no behavioral and neuroimaging research has specifically investigated the effect of gender so far. In a behavioral study by Craik and colleagues, no gender differences with respect to the cognitive protective effect of lifelong bilingualism were found [31]. However, whether males and females differ in the underlying brain areas of the cognitive protective effect of lifelong bilingualism is still unclear. Therefore, future research should take the gender difference better into account and directly investigate it with behavioral and neuroimaging measurements, particularly if one wants to use foreign language learning as a kind of treatment method in enhancing cognitive reserve in aging and delaying the onset and or stages of dementia [138].

Another important issue warranting more research is the relationship between multilingualism, as opposed to bilingualism, and protection against cognitive decline and protection against or delay in the onset of dementia [30]. Differences between multilingual speakers and bilingual speakers might exist in various domains [139] as a multilingual speaker has to switch between more languages and has to suppress and control more languages than a bilingual speaker. In one cross-sectional, multilingualism study controlling for education and age [140], the fact that individuals spoke various languages was more protective than being bilingual. Taken together, learning to speak multiple languages might have a stronger effect on cognitive decline and on the onset or prevention of dementia than being bilingual; however, drawing any firm conclusions on this issue would be premature, and more comprehensive and more appropriate data are needed [141].

Because of the large variability in methodology between the existing bilingualism studies on older adults and patients [142] and the heterogeneity of the bilingual (patient) groups [143], we were of the opinion that it was more useful to investigate which factors play a role in the manifestation of the bilingual advantage. However, one could argue that it would have been better to conduct a meta-analysis that combined individual effect sizes into an average in order to come to a quantitative result [144,145], and to be able to draw a stronger and more objective conclusion about the existence of a possible bilingual advantage.

Another limitation of the present study is that we relied on the conclusions that were drawn by the authors to determine if a result favored the cognitive reserve-enhancing effects of bilingualism, partly supported that hypothesis, or if there was evidence against it. However, according to Paap and colleagues [128], there is a serious risk in this approach because it makes it difficult in terms of critically analyzing individual studies and furthermore opens their summaries to confirmation biases [146]. Paap and colleagues stress the fact that there is a strong tendency for authors to highlight and focus on the comparisons that worked and to ignore or dismiss those that did not [130,147,148,149]. On the other hand, one could also stress that there are tendencies that dismiss positive findings, therefore, because we conducted an overview of studies, we reported the conclusions from the original articles that were published after peer review.

Furthermore, with the specific key words we used (see Materials and Methods) we had a clear focus on the cognitive reserve hypothesis (e.g., the possible relationship between bilingualism and cognitive decline and on the possible delaying effect of bilingualism in the onset of dementia). However, with other key words we would have perhaps been able to include other studies looking at the bilingual advantage in older adults in general. This less narrow approach would have resulted in a larger number of studies and in more negative results than the mixed results that were found in the present systematic review (for an overview of these results, we refer to Paap [145]).

Finally, patients with dementia and their families suffer from many problems and much pain [150]; moreover, the scientific progress that has been made during the last decades, to define the aetiology of neurodegeneration in dementia and to further improve the treatment of those patients is disappointing [151,152,153]. Therefore, the possible usefulness of foreign language learning and the daily active use of two or more languages as an intervention technique in the aging brain is worth investigating [38]. Perhaps learning a foreign language can contribute to some extent to additional cognitive reserve against dementia and might protect from or delay the onset age of the disorder, which is an encouraging outlook in the context of our aging society.

## 5. Conclusions

We found some evidence for a protective effect of bilingualism against cognitive decline in aging, but the results are mixed. Several factors, such as immigration and individual experiences, seem to affect the extent of the cognitive reserve-enhancing effect of lifelong bilingualism. Moreover, several studies reported delayed onset of dementia in bilingual individuals, but again, the results are not clear. Research groups often use different experimental tasks to assess cognitive functioning in healthy older adults and in patients with dementia; therefore, replication studies are warranted with the same methodology to make direct comparisons of the results among research groups possible. Lifelong bilingualism is a complex individual process, and many factors seem to influence this and need to be investigated further in large longitudinal studies with objective behavioral and neuroimaging measurements before the cognitive reserve-enhancing effect of lifelong bilingualism and the protection against dementia is proven.

## Figures and Tables

**Table:** (**a**)

Authors/Publication Year	Number of Subjects	Type of Measurement	Results	In Support of Cognitive Reserve Hypothesis?	Authors’ Conclusions
Kavé et al., 2008 [29]	814 healthy, older adults: 211 were bilingual, 230 were trilingual, and 373 individuals spoke more than three languages	KCST^1^ and MMSE^2^	The number of languages spoken predicted cognitive test scores. This result could not be explained by other variables, such as age, gender, place of birth, age at immigration, or education. Multilingualism was found to be a significant predictor of cognitive state. The individuals who were better in their foreign language than in their mother tongue on average showed better results than the individuals whose mother tongue was their best language. The effect of the number of languages on cognitive state was significant in both groups.	YES	Evidence was found for a cognitive reserve-enhancing effect of lifelong bilingualism, trilingualism, and especially multilingualism.
Crane et al., 2010 [32]	2520 bilingual older adults without dementia	CASI^3^	Neither the use of spoken nor written Japanese in midlife was found to affect cognitive decline in late life.	NO	No evidence was found for a cognitive reserve-enhancing effect of lifelong bilingualism.
Kousaie and Phillips, 2012 [34]	45 healthy older adults: 20 were bilingual and 25 were monolingual	MoCA^4^, Stroop test	No smaller Stroop interference was found for the healthy older bilingual adults as compared to the healthy older monolingual adults. No effect of bilingualism was found in aging on the Stroop task.	NO	No bilingual advantage was found in older adults, questioning the robustness and/or specificity of the cognitive reserve-enhancing effect of lifelong bilingualism.
Bak et al., 2014 [38]	853 healthy older adults: 160 knew 2 languages, 61 knew 3, 16 knew 4, and 8 knew 5	Letter-number sequencing, Matrix reasoning, Block design, Digit symbol, Symbol search, Digit span backward, Logical memory, Spatial span, Moray House Test, NART^5^, and verbal fluency	A beneficial effect of bilingualism on cognition in aging was found, affecting the domains of reading, verbal fluency, and general intelligence more than the domains of memory, reasoning, and speed of processing. No effect of age of acquisition was found. These results cannot be explained by gender, socioeconomic status, or immigration.	YES^6^	Evidence was found for a cognitive reserve-enhancing effect of lifelong bilingualism and multilingualism, even after controlling for childhood intelligence. No effect of age of acquisition was found.
Ihle et al., 2016 [46]	2812 healthy older adults: 1884 spoke one language, 492 two, 281 three, 115 four, 31 five, and 9 six	Mill Hill vocabulary scale, TMT^7^, and interview	The number of languages spoken was found to be a better predictor of cognitive performance than leisure activities and physical demand of job/gainful activity. Educational attainment and cognitive level of job were as good as predictors of cognitive performance.	PARTIAL	Speaking different languages on a regular basis may contribute to cognitive reserve in old age, yet this may not be universal.
Estanga et al., 2017 [48]	278 healthy middle-aged adults: 100 were monolingual, 81 were early bilingual, and 97 were late bilingual	Cerebrospinal fluid AD^8^ markers, MMSE, FCSRT^9^, Digit span test, Stroop test, TMT, verbal fluency, BNT, JLO^10^, 15 object test, and ROCF^11^	A moderation effect was found for bilingualism on both the relationship between age and the presence of AD biomarkers in cerebrospinal fluid and on the relationship between age and executive functioning. Early bilingualism was found to be associated with a better profile of AD biomarkers in cerebrospinal fluid.	YES	Bilingualism contributes to cognitive reserve. It enhances executive and visual-spatial functioning.
Anderson et al., 2018 [50]	61 healthy older adults: 31 were bilingual and 30 were monolingual	Diffusion tensor imaging, MMSE	After controlling and matching for confounds (e.g., intelligence, mini-mental state scores, and demographic variables), a greater axial diffusivity in the left superior longitudinal fasciculus was found in bilinguals compared to monolinguals, indicating a neural reserve in bilingual older adults.	YES	A greater axial diffusivity in the left superior longitudinal fasciculus was found in bilingual older adults compared to monolingual older adults, supporting the cognitive reserve hypothesis.
Mukadam et al., 2018 [51]	2087 healthy older adults: 193 were bilingual and 1894 were monolingual	MMSE, NART, Boston naming test, and verbal fluency	Bilingual older adults had lower MMSE scores than monolingual older adults. This result was entirely explained by education, which also partly explained differences between the two groups in baseline executive functioning. No differences between bilingual older adults and monolingual older adults were found in MMSE decline over time or on baseline tests of executive function.	NO	The authors conclude that bilingualism is a complex phenomenon. When bilingualism is not the result of greater educational attainment, it does not always protect older individuals from cognitive decline.

^1^ KCST = Katzman et al.’s cognitive screening test [62], ^2^ MMSE = Mini-Mental State Examination [63], ^3^ CASI = Cognitive Abilities Screening Instrument [77], ^4^ MoCA = Montreal Cognitive Assessment Test [78], ^5^ NART = National Adult Reading Test [66], ^6^ Note that in contrast to Bak et al. [38], Paap et al. [67] consider their data rather as partial evidence, ^7^ TMT = Trail Making Test [69], ^8^ AD = Alzheimer’s disease, ^9^ FCSRT = Free and Cued Selective Reminding Test [73], ^10^ JLO = Judgement of Line Orientation test of Benton [74], ^11^ ROCF = Rey‒Osterrieth Complex Figure copy [76].

**Table:** (**b**)

Authors/Publication Year	Number of Reviewed Studies	Main Results	Authors’ Conclusions
Bialystok et al., 2016 [53] ^1^	No information given	Bilingualism was found to have protective effects across the lifespan. Bilingual individuals outperformed monolinguals on executive functioning tasks and selective attention tasks.	The results show that bilingualism is a potent source of cognitive reserve.
Quinteros Baumgart and Billick, 2018 [54]	No information given	The results showed that a link exists between bilingualism and higher levels of controlled attention and inhibition in executive control; moreover, bilingualism can protect individuals against the decline of executive control later in life as a result of the increased cognitive reserve. Several factors, like immigration and personal experiences, seem to affect the cognitive reserve-enhancing effect of lifelong bilingualism and multilingualism.	Evidence was found for the cognitive reserve-enhancing effect of lifelong bilingualism and multilingualism. Depending on several factors and individual experiences bilingualism can protect individuals against the decline of executive control in aging.

^1^ This review study taps both aging and cognitive decline and dementia. Therefore, it is listed in both tables, but in the meta-analysis part of this paper, it is only counted once.

**Table:** (**a**)

Authors/Publication Year	Number of Subjects	Type of Measurement	Results	In Support of Cognitive Reserve Hypothesis?	Authors’ Conclusions
Bialystok et al., 2007 [13]	184 patients with dementia: 93 were bilingual and 91 were monolingual	MMSE^1^	The symptoms of dementia appeared 4 years later in the group of older bilingual adults as compared to the group of older monolingual adults. The same results on the MMSE for the bilinguals and the monolinguals were found 4 years prior to the diagnosis of dementia. A shift in onset age of dementia with no change in rate of progression was found in favor of the bilingual older adults.	YES	Evidence was found for the cognitive reserve hypothesis and for the cognitive reserve-enhancing effect of lifelong bilingualism.
Chertkow et al., 2010 [30]	632 patients with probable AD^2^: 253 were multilingual and 379 were monolingual	MMSE	The results showed a protective effect of bilingualism in native Canadians whose first language was French, but not in those whose first language was English. A protective effect of bilingualism was found in immigrants to Canada.	PARTIAL	Overall, lifelong multilingualism (but not bilingualism) was found to have a protective effect.
Craik et al., 2010 [31]	211 patients with probable AD: 102 were bilingual and 109 were monolingual	MMSE	The bilingual patient group showed a later onset of symptoms (5.1 years) and were diagnosed later (on average 4.3 years) than the monolingual patient group.	YES	Lifelong bilingualism was found to be a protective factor against the onset of AD. Support was found for the cognitive reserve hypothesis and the idea of a cognitive reserve-enhancing effect of lifelong bilingualism.
Gollan et al., 2011 [33]	44 bilingual patients with probable AD: 22 were highly educated and 22 were patients with low education	BNT^3^ and subjective rating instrument of second language proficiency	An association was found between higher degrees of bilingualism and increasingly later age-of-diagnosis of AD. The degree of education was found to be an interacting factor. Only objective measures, not self-reported degree of bilingualism, were found to predict age-of-diagnosis of AD.	PARTIAL	Lifelong bilingualism was found to delay the onset of AD, but this was only the case for the patients with a low education level and not for the patients with a high education level. Objective measures, not subjective measures, were found to be predictors.
Sanders et al., 2012 [35]	1779 older adults: 390 were bilingual and 1389 were monolingual	Several language background questions	No association was found between non-native speakers of English and dementia or between non-native speakers of English and AD. When education was assessed further, an increased risk of dementia was found for the non-native speakers of English with more than 16 years of education.	NO	No evidence for a relationship between lifelong bilingualism and the onset of AD was found. A relation might exist in an education-dependent manner, but then in the opposite direction; highly educated bilinguals might be at increased risk.
Schweizer et al., 2012 [36]	40 older adults with probable AD: 20 were bilingual and 20 were monolingual	Analysis of CT^4^ scans	Substantially greater amounts of brain atrophy were found in bilingual patients than in monolingual patients in areas traditionally used to clinically diagnose AD, indicating that greater amounts of neuropathology are needed before the clinical symptoms of AD become visible in bilinguals.	YES	Evidence was found for the cognitive reserve-enhancing effect of lifelong bilingualism and for a delay in the onset of AD in bilinguals.
Alladi et al., 2013 [37]	Case records of 648 middle-aged to older-aged patients with dementia were analyzed: 391 were bilingual and 257 were monolingual	MMSE, ACE-R^5^, and CDR^6^	The bilingual participants developed dementia 4.5 years later than the monolingual participants. This finding could not be explained by other factors, such as education, gender, occupation, living in a city or in the countryside.	YES	Evidence was found for the cognitive reserve hypothesis and for the cognitive reserve-enhancing effect of lifelong bilingualism.
Bialystok et al., 2014 [39]	149 older adults: 76 were bilingual and 73 were monolingual. 74 of the patients had MCI^9^ and 75 had probable AD	MMSE, BNA^7^, D-KEFS^8^	Bilinguals reported later onset ages of the disorder than monolinguals. In the MCI group, the delay was 4.7 years and in the AD group, the delay was 7.3 years in comparison with the monolinguals. These results could not be explained by differences in lifestyle variables, such as smoking, alcohol use, physical activity, diet, or social contacts.	YES	Bilinguals reported later onset ages than monolinguals, supporting the idea that lifelong bilingualism contributes to cognitive reserve. This result could not be explained by differences in lifestyle.
Yeung et al., 2014 [40]	1616 community-living older adults: 703 were bilingual and 913 were monolingual	Structured interview, 3MSE^10^	No association was found between bilingualism and dementia at the first measurement. Also, for the individuals who were cognitively healthy at the first measurement, no association was found between speaking more than one language and dementia at the second measurement five years later.	NO	No association was found between speaking more than one language and dementia.
Zahodne et al., 2014 [41]	1067 older adults: 430 were bilingual and 637 were monolingual. The participants did not initially suffer from dementia	15-item BNT, SRT^11^, WAIS^12^, MDRS^13^, CTT^14^	Although older bilingual adults were found to have better memory and executive function skills at baseline than monolinguals, no protective effect of bilingualism was found among Spanish-speaking immigrants.	NO	No cognitive reserve-enhancing effect of lifelong bilingualism was found. The results show that bilingualism did not alter cognitive decline or protect against dementia.
Kowoll et al., 2015 [42]	86 older adults: 41 were bilingual and 45 were monolingual. 22 of them suffered from MCI and 47 from AD; 17 were healthy controls	MMSE, BNT, TMT^15^, clock drawing test, CERAD-NP^16^, Wechsler memory scale	The study revealed that the dominant language is first affected in bilingual patients with MCI. The bilingual MCI group showed significantly lower verbal fluency and picture-naming scores in their dominant language than bilingual controls. Deficits of the second language appeared later in bilingual patients suffering from AD when compared to bilingual controls.	NO	No cognitive reserve-enhancing effect of lifelong bilingualism was found.
Lawton et al., 2015 [43]	81 older adults with AD: 27 were bilingual and 54 were monolingual	Verbal learning test, SENAS^17^, IQCODE^18^ 3MSE	The bilingual older adults were more highly educated than the monolingual older adults. This was not the case for the U.S. born bilinguals and monolinguals. No differences between the bilinguals and monolinguals were found in the mean age of dementia diagnosis.	NO	No differences in age of onset of AD were found between bilinguals and monolinguals, showing no evidence for a protective effect of lifelong bilingualism.
Woumans et al., 2015 [44]	134 patients with probable AD: 65 were bilingual and 69 were monolingual	MMSE	For the bilingual patients, a delay was found, on average, of 4.6 years in manifestation and 4.8 years in diagnosis compared to the monolingual patients.	YES	Evidence was found for the cognitive reserve hypothesis and for the cognitive reserve-enhancing effect of lifelong bilingualism.
Clare et al., 2016 [45]	86 older adults with probable AD: 37 were bilingual and 49 were monolingual	Background measures, MMSE, a whole test battery of executive functioning tasks	No clear advantage in executive functioning was found in the bilinguals compared to the monolinguals. A delay in AD may exist in bilinguals, but if so, the results are less convincing than in previous studies. The bilingual patients came later to the attention services than the monolingual patients.	NO	A delay in the onset of AD may occur, but if so, the results are less convincing than the previously reported results in the literature.
Kowoll et al., 2016 [47]	30 older adults: 16 were lifelong bilingual and 14 were monolingual. 12 were diagnosed with MCI and 18 with early stage AD	FDG^19^ and PET^20^	The results showed that the bilingual patients showed substantially greater impairment of glucose uptake in frontotemporal regions, patietal regions, and in the left cerebellum in comparison with monolingual patients.	YES	Bilingualism is likely to contribute to cognitive reserve on a neural level.
Perani et al., 2017 [49]	85 patients with probable AD: 45 were bilingual and 40 were monolingual	Brain metabolism and neural connectivity	An increased connectivity in the executive control and in the default mode networks was found in the bilingual patients compared to the monolingual patients. The degree of lifelong bilingualism (i.e., high, moderate, or low use) was found to significantly correlate to functional modulations in crucial neural networks.	YES	Evidence was found for both neural reserve and compensatory mechanisms in bilingual patients with probable AD, supporting the cognitive reserve-enhancing effect of lifelong bilingualism.
Zheng et al., 2018 [52]	129 older adults with probable AD: 61 were bilingual and 68 were monolingual	Structured interview, MMSE	The results showed that the Cantonese/Mandarin bilinguals had a delay in onset of AD of 5.5 years and, furthermore, visited the clinic later compared to the monolinguals.	YES	Constantly speaking two languages from at least early adulthood can delay the onset of AD, supporting the cognitive reserve hypothesis.

^1^ MMSE = Mini-Mental State Examination [63], ^2^ AD = Alzheimer’s disease, ^3^ BNT = Boston Naming Test [84], ^4^ CT = computed tomography, ^5^ ACE-R = Addenbrooke’s Cognitive Examination-Revised [37], ^6^ CDR = Clinical Dementia Rating [37], ^7^ BNA = Behavioral Neurology Assessment [81], ^8^ D-KEFS = Delis–Kaplan Executive Function System Tests [82], ^9^ MCI = Mild cognitive impairment, ^10^ 3MSE = Modified Mini-Mental State Examination [83], ^11^ SRT = Selective Reminding Test [85], ^12^ WAIS = Wechsler Adult Intelligence Scale‒Revised [86], ^13^ MDRS = Mattis Dementia Rating Scale [87], ^14^ CTT = Color Trails Test [88], ^15^ TMT = Trail Making Test [69], ^16^ CERAD‒NP = consortium to establish a registry for Alzheimer’s disease – neuropsychological test battery [42], ^17^ SENAS = Spanish and English Neuropsychological Assessment Scales [93], ^18^ IQCODE = the Informant Questionnaire on Cognitive Decline in the Elderly [94], ^19^ FDG = Fludeoxyglucose, ^20^ PET = Positron emission tomography.

**Table:** (**b**)

Authors/Publication Year	Number of Reviewed Studies	Main Results	Authors’ Conclusions
Freedman et al., 2014 [55]	4 original studies	One Canadian (Toronto) and one Indian (Hyderabad) study showed a significant effect of lifelong bilingualism in delaying the onset of AD by up to 5 years whereas another Canadian study (Montreal) showed this effect only for multilingual individuals who speak at least four languages or for immigrants who speak at least two languages.	A protective effect of bilingualism in delaying onset of dementia was found. In the context of specific cultural and immigration factors, only multilingualism, not bilingualism, leads to a postponement of dementia. This needs to be investigated further in future cross-cultural studies.
Gold 2015 [56]	No information given	The protective and delaying effect of bilingualism against the symptoms of AD may work via the frontostriatal and frontoparietal executive functioning networks rather than medial temporal lobe memory networks. In addition, the beneficial effects of bilingualism to cognitive reserve may work via specific cellular and molecular mechanisms.	Evidence exists in the literature for a delay of the onset of AD symptoms in bilingual older adults by several years.
Guzmán-Vélez et al., 2015 [57]	15 original studies	Lifelong bilingualism was found to be related to more efficient use of brain resources, helping bilingual individuals to maintain cognitive functioning in the presence of neuropathology. The authors discuss several neural mechanisms underlying this phenomenon.	Evidence was found for the idea that lifelong bilingualism is a cognitive (and possibly brain) reserve enhancing factor. More research on the relationship between bilingualism, education, and the onset of dementia is warranted. This might help individuals in the prevention of and/or coping with a brain disease in a better way in the future.
Perani and Abutalebi 2015 [58]	No information given	The use of two or more languages was reported to affect the human brain in terms of anatomo-structural changes. A significant delay of dementia onset was found in bilingual/multilingual individuals. This result was found in different studies conducted in different countries and with different cultural backgrounds of the individuals.	Lifelong bilingualism was found to be a powerful cognitive reserve factor. The onset of dementia in bilingual individuals is delayed by approximately 4 years as compared to monolingual individuals. Lifelong bilingualism results in increases of gray and white matter, especially when frequent second language exposure and use is present throughout life.
Bialystok et al., 2016 [53] ^1^	No information given	A 4- to 5-year delay in onset age of dementia was found in retrospective studies for bilingual older adults compared to monolingual older adults. These results could not be explained away by factors such as immigration, education, socio-economic background, and age of second language acquisition.	The results showed a protective effect of bilingualism against symptoms of dementia. In general, a delay of between 4 and 5 years in the onset age of dementia was found.
Calvo et al., 2016 [59]	17 original studies	Interpreting the results on the possible relationship between bilingualism and cognitive reserve has been difficult so far. More stringent control of relevant variables is needed. The focus is only on the delay of AD, instead of the changes during the different stages of the disease.	A better methodology in the studies on the relationship between bilingualism and cognitive reserve is needed in order to draw any firm conclusions about the unique cognitive reserve contribution of bilingualism in patients with AD at the different stages of the disease.
Klimova et al., 2017 [60]	14 original studies	Bilingualism was found to delay the onset of dementia in retrospective studies, but this result was not confirmed in prospective studies. More research on the relationship between bilingualism and a delay in the onset of dementia is warranted, especially because positive findings were found in brain studies that investigated the relationship between bilingualism and cognitive reserve.	Evidence was found for the contribution of bilingualism to cognitive reserve in retrospective studies, but this result was not confirmed in prospective studies. Methodological weaknesses in the retrospective studies seem to explain the different findings.
Mukadam et al., 2017 [61]	13 original studies included in qualitative synthesis, of which 4 were included in the meta-analysis	The prospective studies showed no evidence that bilingualism protects against cognitive decline or dementia. Retrospective studies show a different picture, supporting the hypothesis that it contributes to cognitive reserve, protects against cognitive decline, and delays the onset of dementia. These beneficial effects of bilingualism in retrospective studies are affected by differences in education and culture. Therefore, these studies give no insight into the causative relations.	The results obtained in retrospective studies show support for the cognitive reserve hypothesis and for the cognitive reserve-enhancing effect of lifelong bilingualism, but the results obtained in prospective studies do not. Retrospective studies are not suitable to provide any information about the causative relations between bilingualism and cognitive reserve.

^1^ This review study taps both aging and cognitive decline and dementia. Therefore, it is listed in both tables, but in the meta-analysis part of this paper, it is only counted once.
